# Evaluation of Toxicity of Crude Phlorotannins and Phloroglucinol Using Different Model Organisms

**DOI:** 10.3390/toxins14050312

**Published:** 2022-04-28

**Authors:** Dicky Harwanto, Bertoka Fajar Surya Perwira Negara, Gabriel Tirtawijaya, Maria Dyah Nur Meinita, Jae-Suk Choi

**Affiliations:** 1 Seafood Research Center, Industry Academy Cooperation Foundation (IACF), Silla University, 606, Advanced Seafood Processing Complex, Wonyang-ro, Amnam-dong, Seo-gu, Busan 49277, Korea; ftrnd12@silla.ac.kr (B.F.S.P.N.); tirtawijayag@yahoo.com (G.T.); 2 Faculty of Fisheries and Marine Science, Diponegoro University, Semarang 50275, Indonesia; 3 Department of Marine Science, University of Bengkulu, Jl. W.R Soepratman, Bengkulu 38371, Indonesia; 4 Faculty of Biotechnology, University of Surabaya, Jalan Raya Kalirungkut Surabaya, Surabaya 60292, Indonesia; 5 Faculty of Fisheries and Marine Science, Jenderal Soedirman University, Purwokerto 53123, Indonesia; 6 Center for Maritime Bioscience Studies, Jenderal Soedirman University, Purwokerto 53123, Indonesia; 7 Department of Food Biotechnology, College of Medical and Life Sciences, Silla University, 140, Baegyang-daero 700 beon-gil, Sasang-gu, Busan 46958, Korea

**Keywords:** *Ecklonia cava*, algae, seaweed, invertebrate, allelopathy, phlorotannins

## Abstract

Phlorotannins have been proven to contain numerous bioactive compounds that have potential to be applied in variety industries, including cosmetics, functional foods, nutraceuticals, environmental management, and medicine. The larvicidal and growth-inhibiting properties of phlorotannins have been extensively studied in various organisms. However, the toxicity of the phloroglucinol oligomer of phlorotannin is unclear, especially in *Artemia salina*, *Daphnia magna*, *Lactuca sativa*, and *Chlorella vulgaris*, which are commonly used in many bioassays. Therefore, research using these four organisms should be designed to provide basic information about the toxic effects of phlorotannins and phloroglucinol. This study aimed to evaluate the larvicidal and inhibitory properties of phlorotannins and phloroglucinol on *A. salina*, *D. magna*, *L. sativa*, and *C. vulgaris*. Phlorotannin extract and phloroglucinol were administered at various concentrations to each test organism. The survival rate of *A. salina* nauplii and *D. magna* neonates was observed every 24 h to 72 h, whereas the *L. sativa* seed germination and inhibition rate of *C. vulgaris* were observed up to 96 h. The results showed that the 24 h LC50 of phlorotannin on *A. salina* and *D. magna* were 10.67 and 1.32 mg/mL, respectively. The germination inhibition of *L. sativa* was 53.3% with a seed growth of less than 4 mm after 96 h upon exposure to 1 mg/mL of phlorotannin. Freshwater and seawater *C. vulgaris* experienced yield inhibition of 39.47 and 43.46%, respectively, when 2 mg/mL of phlorotanin was added. These results indicate that phlorotannin affects the survival and growth of the test organisms, so its use as a pesticide, herbicide, and algaecide agent for environmental and aquaculture applications can be further studied.

## 1. Introduction

Phlorotannins have been isolated from brown seaweeds, including *Ecklonia cava* [1,2,3,4], *E. stolonifera* [5], *Sargassum ringgoldianum* [6], *Ishige okamurae* [7], *Fucus vesiculosus* [8,9], and *Eisenia bicyclis* [10]. Phlorotannins are highly hydrophilic compounds formed by the acetate-malonate pathway. They contain phloroglucinol units (1,3,5-trihydroxybenzene), have a molecular size of 126 Da–650 kDa, and a concentration of 0.5% to 2.5% (w/w) in dried brown seaweeds [11,12].

Previous studies have investigated the presence of numerous biological activities of phlorotannin with pharmacological application values, such as antioxidant [7], anti-inflammatory [13], anti-cancer [14], anti-diabetic, and neuro-protective [15] properties. Phlorotannins are also toxic to other organisms. Recent scientific reviews of the toxicity and larvicidal activity of phlorotannins in organisms have been compiled [16,17]. Larvicidal studies of phlorotannins in mosquito larvae have also been widely reported [18,19,20], as well as in some marine invertebrates, such as ascidians *Ciona savignyi* and *Halocynthia roretzi* [21]; tube-building polychaete, *Hydroides elegans* [22]; mussel, *Mytilus edulis* [23]; blue crab, *Portunus trituberculatus* [24]; barnacle, *Balanus improvisus* [9]; coral, *Acropora millepora* [25]; and brine shrimp, *Artemia salina* [26]. Studies on the growth inhibitory effect of phlorotannins have included the green algae *Enteromorpha prolifera* [23] and microalgae associated with red tides (*Chattonella antiqua* and *Cochlodinium polykrikoides*) [24]. The effect of phlorotannin on several types of epiphyte macroalgae has been examined [27]. The toxicity of phlorotannin in the germination of lettuce (*Lactuca sativa*) has been assessed [28].

Phloroglucinol, which is structurally related to phlorotannins, is believed to be harmful to organisms. Lau and Qian [22] investigated the toxic effects of phloroglucinol in *H. elegans* larvae. However, data remain scant. The toxicity of phloroglucinol to *A. salina, L. sativa*, *Chlorella vulgaris*, or *Daphnia magna* is unknown. Indeed, the toxicity of phlorotannin on *D. magna* has not been described.

Some species of brown seaweed have been investigated as sources of phlorotannin extracts and have been tested for their toxicity on invertebrates, microalgae, seaweed, and plants. However, from the many types of brown algae reported, there has been no scientific report on the toxic effect of phlorotannin extracted from *E. cava* against *D. magna*, *L. sativa*, and *C. vulgaris*. Although phlorotannins extracted from different seaweed sources contain the same monomer (phloroglucinol unit), there may be some differences in their bioactivity and toxicity. This is due to its structural composition, additional modifications, and molecular weight [29]. The characterization and quantitative analysis of phlorotannins compounds have been widely reported [30,31,32].

*E.cava* is abundant in Korean waters and has been used as a food ingredient and herbal medicine for generations. However, its potential use concerning environmental management is still less known. Therefore, knowledge of the larvicidal and allelopathic properties of phlorotannin extracted from E. cava, and also phloroglucinol in several model organisms, namely *D. magna*, *A. salina*, *L. sativa*, and *C. vulgaris*, is important. Hence, the purpose of this study was to investigate the lethal and inhibitory concentration of phlorotannin extracted from E. cava and phloroglucionol on *D. magna*, *A. salina*, *L. sativa*, and *C. vulgaris*. This information can be used to develop phlorotannin and phloroglucinol as a natural pesticide, herbicide, and algaecide in water environment and aquaculture.

*Artemia* sp. is one of the most frequently used invertebrate species for toxicity testing in seawater [33,34,35,36], while for toxicity testing in freshwater, *Daphnia* sp. is the most commonly used [37,38,39,40,41,42,43]. The tendency of those species as the main choice for toxicity testing is related to several factors, including the relatively short duration of observation, relatively low cost, good knowledge of their biology and ecology, relatively easy manipulation and maintenance in the laboratory, small body size allowing accommodation in small beakers or microplates, and high adaptability to numerous test conditions [33,34,35,36]. Lettuce is a very popularly used plant for growth inhibition assays [28,44,45,46,47,48]. Its relatively easy and fast growth makes it one of the main choices for toxicology testing. Microalgae are important organisms for monitoring water quality, as they are at the lowest level of the food chain in aquatic ecosystems. The cell density (CD) and daily cell weight (DCW) measurements are commonly used to indicate microalgae growth, as has been performed in previous studies on *C. vulgaris* [49,50,51,52,53] and other species, such as *Chlamydomonas mexicana* and *Scenedesmus obliquus* [54]. Furthermore, the yield inhibition rate is widely used to evaluate the inhibitory effect of environmental pollutants on algal growth as a comprehensive factor for determining the phytotoxicity of pollutants to algae [53,55,56].

The purpose of this study was to investigate the toxic effects of various concentrations of phlorotannins and phloroglucinol on invertebrate larvae, plants, and microalgae. Nauplii of *A. salina* and neonates of *D. magna* were used as seawater and freshwater invertebrates representatives, respectively. Freshwater and seawater *C. vulgaris* were used as the algae test, representing seawater and freshwater microalgae and *L. sativa* as the representative plant. The results provide important information for future research and environmental applications of phlorotannin or phloroglucinol as natural pesticides, algaecides, and herbicides in aquaculture. Natural pesticides, algaecides, and herbicides have advantages such as being environmentally friendly, biodegradable, and harmless to non-targeted organisms.

## 2. Results

### 2.1. Phloroglucinol

**Figure 1:**
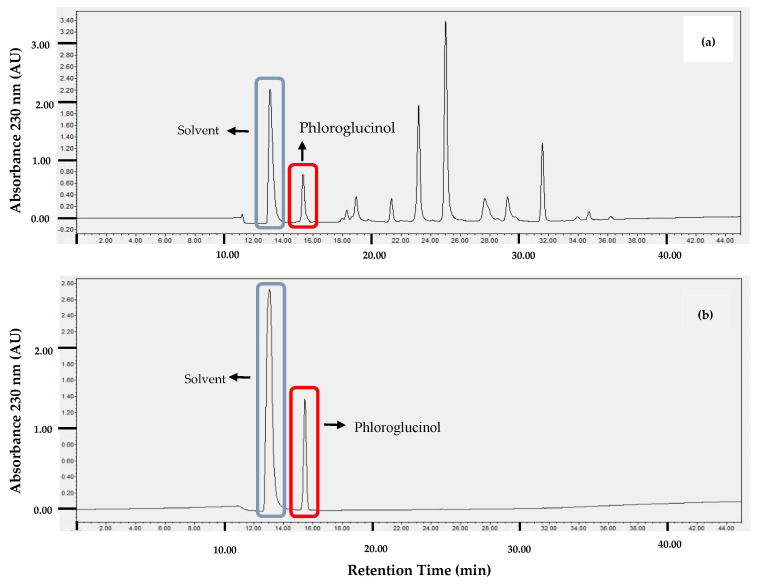
Profile phloroglucinol: (**a**) crude phlorotannins profile (5 mg/mL) and (**b**) standard phloroglucinol (0.5 mg/mL).

Phlorotannins consist of polymerized phloroglucinol (1,3,5-trihydroxybenzene) in divers forms, with different amounts of phloroglucinol and/or a different type of bond. The phloroglucinol retention time in the present study was 15.3–15.5 min (Figure 1a,b). Five grams of crude phlorotannin extract contained 0.4797 mg phloroglucinol (9.59%).

### 2.2. Larvicidal Effects of Crude Phlorotannin and Phloroglucinol on Survival of Artemia salina Nauplii

Pure seawater used as a negative control did not kill *A. salina* nauplii after 24 h and 48 h (Table 1), nor did methanol at a concentration of 2.5% as a control vehicle. Thus, 2.5% methanol was not toxic to *A. salina* [26]. Death of *A. salina* nauplii was evident at 72 h. However, the mortality rate was still relatively low (10% and 11% in the negative control and control vehicle, respectively).

The larvicidal effects of four different concentrations of crude phlorotannin on *A. salina* nauplii larvae are shown in Table 1. Survival rates of *A. salina* exposed to phlorotannin extract at concentrations of 0.1, 1.0, and 5.0 mg/mL were still 100% at 24 h but were 81.3% at 10.0 mg/mL and 0.0% at 50.0 mg/mL. In this period, all extracts with different concentrations were still classified as non-larvicidal, except at 50.0 mg/mL, which was extremely larvicidal. Based on the Probit analysis, the 24 h LC50 was 10.67 mg/mL. After 72 h, the concentration of 10.0 mg/mL showed a relatively low survival rate value of 37.5%; thus, it was classified as mildly larvicidal, with a 72 h LC50 of 6.50 mg/mL. 

Five different concentrations of phloroglucinol were tested on *A. salina* nauplii (Table 1). The two lowest concentrations (0.005 and 0.01 mg/mL phloroglucinol) were non-larvicidal for *A. salina* after 72 h of observation. The 0.05 mg/mL concentration showed no larvicidal effect at 24 h (68.3% survival). A mild larvicidal effect was evident at 48 h (48.3% survival) and at 72 h (31.7% survival). At 0.1 mg/mL, phloroglucinol was extremely larvicidal. The survival rate for *A. salina* was only 1.7% at 24 h, after which the mortality was 100.0%. At the highest concentration of 0.5 mg/mL, phloroglucinol was extremely larvicidal to *A. salina*, with 100.0% mortality at 24 h. The LC50s were 59.72 and 26.40 µg/mL at 24 h and 72 h, respectively.

### 2.3. Larvicidal Effect of Crude Phlorotannin and Phloroglucinol on Survival of Daphnia magna Neonates

The use of 2.5% methanol as a vehicle for *D. magna* neonates was nontoxic (Table 2), as evident by the high survival rates at 24, 48, and 72 h of exposure (92.5%, 85.0% and 85.0%, respectively). As expected, the pure seawater negative control also showed high survival rates of 95.0%, 87.5%, and 87.5% at 24, 48, and 72 h, respectively.

The larvicidal effect of 0.5, 1.0, and 2.0 mg/mL phlorotannin was examined in *D. magna* neonates. All concentrations tested showed larvicidal activity (Table 2). The survival rates of neonates after exposure to 0.5 and 1.0 mg/mL for 24 h were 91.3% and 63.8%, respectively, and were considered non-larvicidal. In contrast, the survival rate of 16.3% using the 2.0 mg/mL concentration was highly larvicidal (24 h LC50 of 1.32 mg/mL). This concentration was extremely larvicidal at 48 h, with 100.0% mortality. The 5.0 mg/mL concentration was mildly larvicidal after 72 h of exposure (31.3% survival). The 1.0 mg/mL concentration was highly larvicidal (7.5% survival; 72 h LC50 was 278.74 µg/mL).

Phloroglucinol levels at 0.005, 0.01, and 0.05 mg/mL were not larvicidal for *D. magna* neonates during 72 h of observation (Table 2). The concentration of 0.1 mg/mL showed mild larvicidal effects at 24 and 48 h with survival rates of 28.3% and 25.0%, respectively. This concentration was highly larvicidal at 72 h with a survival rate of only 16.7%. At 0.5 mg/mL phloroglucinol, the effect was extremely larvicidal, with no survival within 24 h. The 24 h and 72 h LC50s were 68.91 and 60.25 µg/mL, respectively. 

### 2.4. Inhibitory Effect of Crude Phlorotannin and Phloroglucinol on Germination of Lactuca Sativa Seed 

The allelopathic potential of various concentrations of crude phlorotannin from *E. cava* was tested based on the germination and growth of *L. sativa* seedlings. All three extracts inhibited the germination of *L. sativa*. At concentrations of 1, 10, and 50 mg/mL, seeds that germinated after the 24 h incubation period were only 40.0, 2.5, and 2.5%, respectively (Table 3). At 48 h, germinating seeds increased to 57.5, 37.5, and 17.5%, respectively. Up to the 96-h period, the seeds that germinated were 70.0, 70.0, and 52.5%, respectively, while that in the vehicle was 95.0%.

Three of the five concentrations of phloroglucinol tested (50, 100, and 500 µg/mL) showed no inhibitory effect on the germination of *L. sativa* seeds (Table 3). The 1000 µg/mL concentration displayed an inhibitory activity, with a germination rate of 53.3% and an average seed growth of <4 mm at the end of the observation. The highest concentration of 5000 µg/mL was absolutely lethal to *L.sativa*, with a seed germination rate of 0%.

### 2.5. Inhibitory Effect of Crude Phlorotannin and Phloroglucinol on Freshwater Chlorella vulgaris

**Figure 2:**
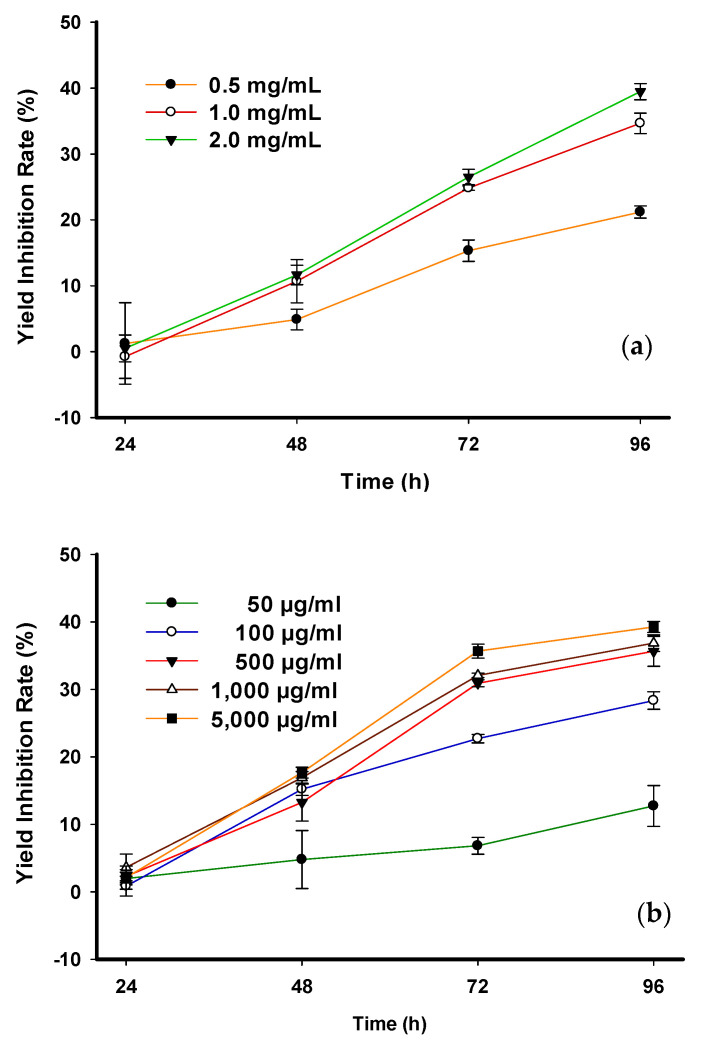
Toxicity of (**a**) crude phlorotannin and (**b**) phloroglucinol on yield inhibition rate (%) of freshwater *Chlorella vulgaris*.

The CD of the control group did not increase significantly compared with all treatment groups at 24 and 48 h, with the exception of the highest concentration after 48 h (Table 4). Significant differences were observed at 72 h when the control group increased more sharply than the others. The control group obviously increased much higher than others, so more significant differences were seen at 96 h. The rates of yield inhibition in all treatment groups versus that in the control groups increased with increasing exposure time (Figure 2a). On the last day, the group treated with the highest concentration of phlorotannin displayed the greatest inhibition and vice versa. The rates of yield inhibition for the 0.5, 1.0, and 2.0 mg/mL concentrations of phlorotannin were 21.21%, 34.67%, and 39.47%, respectively, after 96 h.

At 48 h, only the control and 50 µg/mL phloroglucinol groups had increased CD. The CD control was not significantly higher in the 50 µg/mL treatment but was significantly higher than that of the other treatments. The CD in the control group increased significantly at 72 h and 96 h compared to that in all treatment groups. The yield inhibition rates in all treatment groups decreased with increasing exposure times compared to the control group (Figure 2b). At 96 h, the yield inhibition rates of freshwater *C. vulgaris* groups treated with 50, 100, 500, 1000, and 5000 µg/mL of phloroglucinol were 12.72%, 28.36%, 35.66%, 36.86%, and 39.27%, respectively.

### 2.6. Inhibitory Effect of Phlorotannin and Phloroglucinol on Seawater Chlorella vulgaris

**Figure 3:**
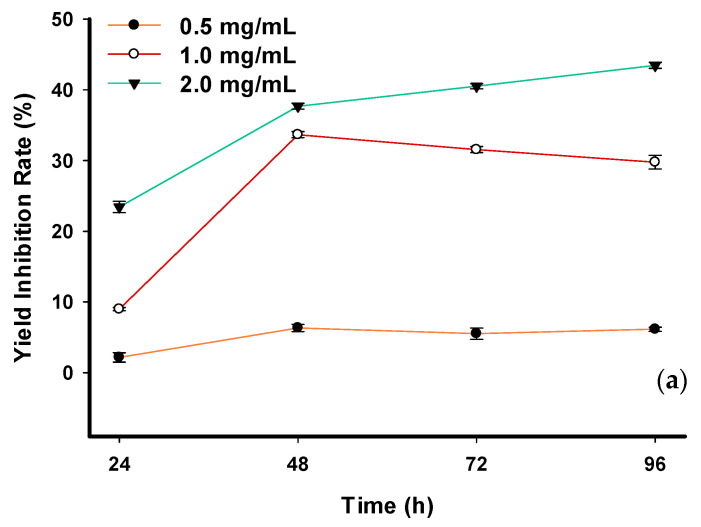

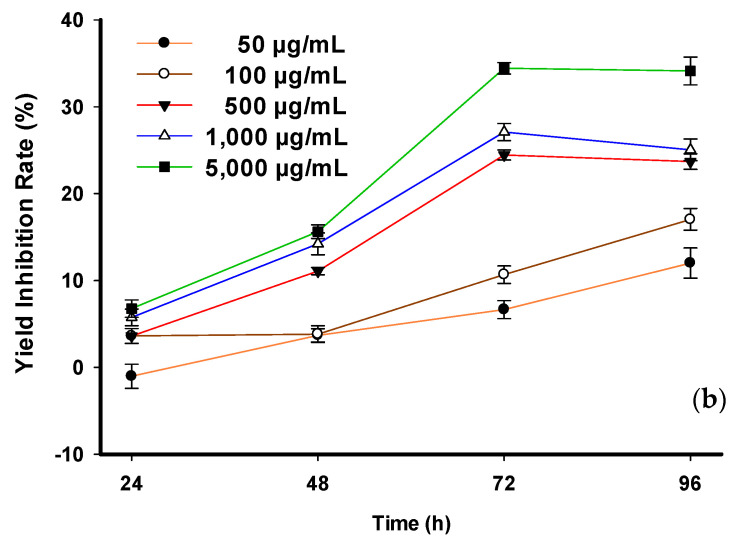
Toxicity of (**a**) crude phlorotannin and (**b**) phloroglucinol on yield inhibition rate (%) of seawater *Chlorella vulgaris*.

Seawater *C. vulgaris* in the control group and the group treated with 0.5 mg/mL phlorotannin showed growth, as evidenced by an increase in CD (Table 5). In contrast, the 1.0 mg/mL treatment group showed fluctuations, and the 2.0 mg/mL group displayed decreases. After 24 h, the CDs of the control group were not significantly different from those of the lowest concentration treatment group but were significantly different from those of the other treatments. At 72 h and 96 h, the CDs of the control group were significantly different from those of the other three groups. The yield inhibition rate of the three treatment groups decreased with increasing exposure time (Figure 3a). At 96 h, the highest yield inhibition rate of 43.46 ± 0.43% occurred in the group treated with the highest concentration of phlorotannin. The lowest value of 6.15 ± 0.31% was observed in the group treated with the lowest concentration of phlorotannin.

The control group and the three treatment groups showed an increasing trend in CD from 0 to 96 h (Table 5). A slight decrease occurred at 24 to 48 h in all treatment groups, except at 100 µg/mL. Subsequently, all groups experienced an increase until the end of the observation period. Starting at 24 h, the CD values of the control group increased significantly for all groups, except for the 50 µg/mL group, which started at 48 h. The yield inhibition rate of all treatment groups decreased with the duration of phloroglucinol exposure, except in the lowest treatment group, which increased slightly at the end of the observation period (Figure 3b). From 24 to 96 h, the group treated with the highest concentration of phloroglucinol displayed the greatest yield inhibition rate, with the value at the end of observation being 34.14 ± 1.60%.

## 3. Discussion

### 3.1. Larvicidal Effect of Crude Phlorotannin and Phloroglucinol on Survival of Artemia salina Nauplii

In this study, phlorotannin concentrations up to 10 mg/mL (1.0%) were not larvicidal until 48 h (24 h LC50 of 10.67 mg/mL) against *A. salina* nauplii. A larvicidal effect occurred at 72 h of exposure to 1.0% phlorotannin, with >50% mortality. For phloroglucinol, mortality exceeded 50% at a concentration of 0.1 mg/mL at 24 h (24 h LC50 of 59.72 µg/mL). After a longer exposure period (48 h), a mortality >50% also occurred at a lower concentration (0.05 mg/mL).

The larvicidal activity of phlorotannin extracted from *E. cava* on the survival of *A. salina* nauplii was comparable to that of phlorotannins extracted from other seaweeds. Kim and Choi [26] investigated the toxicity of phlorotannin, which was extracted from 13 seaweeds. They reported that phlorotannins extracted from 13 brown seaweeds showed larvicidal activity with a mortality > 50% against *A. salina* nauplii at a concentration of 2.5% after 48 h of exposure.

Ayesha et al. [57] screened the cytotoxic activity of several brown algae species found at Karachi beach on *A. salina* nauplii. After 24 h, ethanol extracts of the brown seaweeds displayed different toxicity potentials. *Sargassum ilicifolium* was the weakest (LC50 1000 µg/mL), followed by *Dictyota dichotoma* var. *velutricata* (LC50 812 µg/mL), *Jolyna laminarioides* (LC50 812 µg/mL), *Sargassum lanceolatum* (LC50 800 µg/mL), *Dictyota hauckiana* (LC50 524 µg/mL), *Iyengaria stellata* (LC50 186 µg/mL), and *Drosera indica* (LC50 141 µg/mL). The mortality of nauplii increased with increasing extract concentration. 

The larvicidal effects of various brown algae extracts on marine invertebrates have been widely investigated. The chemical inhibition of barnacle larvae settlement by the brown algae *F. vesiculosus* has been reported [9]. The authors showed that water containing 31.5 μg/mL phlorotannins from *F. vesiculosus* inhibited the settlement of barnacle larvae. Birrel et al. [25] investigated the chemical effects of *Padina* sp. On the larval settlement of the broadcast-spawning coral *A. millepora*. Larval settlement in seawater collected from tanks containing the closely related alga *Padina* sp. was almost one-third less than in the substratum control, i.e., 39%.

### 3.2. Larvicidal Effect of Crude Phlorotannin and Phloroglucinol on Survival of Daphnia magna Neonates 

*Daphnia magna* neonates are popularly used as test animals in aquatic toxicity assays by placing them in a test vessel with a density of five neonates per well containing 10 mL of solution [58,59,60]. In this study, 20 *D. magna* neonates were placed in a relatively small container in aerated 24-well plates. Each well contained 2 mL of solution. The use of a relatively small volume of solution allows for the minimal use of bioactive compounds and reagents. 

A recent study observed that a phlorotannin concentration of 2 mg/mL extracted from *E. cava* had high larvicidal activity against *D. magna* neonates (24 h LC50 of 1.32 mg/mL). Furthermore, even a concentration of 0.5 mg/mL was able to kill >50% of the population within 48 h. The same larvicidal effect was demonstrated with the use of phloroglucinol at a much lower concentration of 0.1 mg/mL (24 h LC50 of 68.91 µg/mL).

The larvicidal effect of brown seaweed extracts on *D. magna* has not been reported. However, several studies have involved freshwater mosquito larvae. Phlorotannin extract from the brown algae *D. dichotoma* was reportedly the most effective against *Aedes aegypti*, with an LC50 of 61.66 mg/L at 24 h [18]. Manilal et al. [19] demonstrated that phlorotannin extracts from *Lobophora variegata*, *Stoechospermum marginatum*, and *Sargassum wightii* could also serve as natural larvicidal compounds against the larvae of *A. aegypti* and *Culex quinquefasciatus*. The LC50 values after 24 h of *L. variegata* against *A. aegypti* and *C. quinquefasciatus* were 70.38 µg/mL and 79.43 µg/mL; those of *S. marginatum* were 82.95 and 85.11 µg/mL; and those of *S. wightii* were 84.82 and 87.09 µg/mL, respectively. The larvicidal activity of extracts derived from *Sargassum binderi* and *Padina australis* seaweed has also been tested on *A. aegypti* larvae [61]. The authors reported that the mortality rate of methanol extract of *P. australis* (LC50 400.46 µg/mL) was 53.33% and that that of *S. binderi* methanol extract (LC50 217.04 µg/mL) was 50.67% at 24 h.

In the present study, *D. magna* displayed increased mortality after exposure to higher concentrations and longer exposure to phlorotannins and phloroglucinol. The chemical composition of seaweeds plays an important role in bioactivity. The larvicidal effects of phlorotannins and phloroglucinol indicate that polyphenols play an important role in this phenomenon. As reported by Yu et al. [61], seaweeds with a higher total polyphenol content have a higher larvicidal effect.

The larvicidal effect of phlorotannin on *D. magna* appeared stronger than on *A. salina*. This is thought to be related to the character of the organism. *Daphnia* sp. is an organism that is more sensitive to environmental toxicity than *Artemia* sp. A similar result was reported by de la Vega et al. [62], where *D. magna* proved to be a sensitive organism, and *Artemia* sp. proved to be a resistant organism when exposed to nTiO_2_ in combination with an organic UV filter.

### 3.3. Inhibitory Effect of Crude Phlorotannin and Phloroglucinol on Germination of Lactuca sativa Seeds 

Phlorotannin showed its allelopathic activity on the germination and growth of *L. sativa*. Up to 96 h of exposure, all groups of *L. sativa* treated with phlorotannin showed a lower percentage of germination and radicle length than did the control (*p* < 0.05). With the addition of phloroglucinol, the percentage of germination and radicle growth showed significant inhibitory activity at concentrations of 0.5 mg/mL (80.0%; 11.73 mm) and 1.0 mg/mL (53.3%; 3.53 mm) compared to the control (95.0%; 25.29 mm). At a higher concentration of 5.0 mg/mL, phloroglucinol showed extreme lethality, in which no seed was able to germinate. 

The present results are consistent with the results of previous studies. Brown seaweed has often been tested as an inhibitor of plant germination. Loliolide and epiloliolide are compounds isolated from *Sargassum crassifolium* and have been tested as inhibitors of head lettuce seed germination [44]. Both compounds produced an inhibition rate > 80% at a concentration of 5 g/cm^2^. Furthermore, complete inhibition was observed at a concentration of 80 g/cm^2^ for both compounds. Fonseca et al. [63] evaluated the effect of *Dictyota menstrualis* acetone extract on the germination of *Mimosa pudica* and *Senna obtusifolia*. The evaluation revealed that the administration of 20 ppm (20 µg/mL) extract had strong inhibitory effects on seed germination, respectively, reaching 88% and 77%. Baroud et al. [64] observed that aqueous extracts of *Cystoseira gibraltarica* and *Bifurcaria bifurcata* at 2% (20 g/L) on tomato (*Solanum lycopersicum*) produced germination inhibition rates of 63% and 42%, respectively. Several studies have reported that brown seaweed acts as a fertilizer at relatively low concentrations. Some selected machair crops experienced increased germination with the addition of 5% extract of kelp (*Laminaria digitata*). In contrast, germination was completely inhibited in meadow buttercups (*Ranunculus acris*) at the same extract concentration [65]. The germination of fenugreek increased in the presence of extract from *S. vulgare* and *Padina pavonica* at concentrations up to 10% (100 g/L) and *Colpomenia sinuosa* extract concentrations up to 15% [66]. However, an inhibitory effect was observed when the concentration of the extract was increased.

### 3.4. Inhibitory Effect of Crude Phlorotannin and Phloroglucinol on Freshwater and Seawater Chlorella vulgaris

The effects of phlorotannin and phloroglucinol on the cell density (CD) of freshwater *C. vulgaris* had no significant effect on any of the treatments at 24 h (*p* > 0.05). Significant effects started at 48 h, except for the lowest concentration, for which effects were observed at 72 h. The growth of seawater *C. vulgaris* was significantly different at 24 h of cultivation in all treatment groups, except for the lowest concentration observed at 48 h of cultivation. The lower biomass of the control in all treatments when phlorotannin or phloroglucinol was added to the medium indicated that the specific concentration had an inhibitory effect on algal growth. The CD growth of *C. vulgaris* was further inhibited by increasing concentrations of phlorotannins and phloroglucinol compared to controls. 

In contrast, the inhibition of the growth rate decreased with an increasing cultivation period. The inhibitory effect weakened with an increasing exposure time. In general, this trend of decreasing inhibition rate occurred in all treatments, for both freshwater and seawater *C. vulgaris*. These findings indicate that *C. vulgaris* has a capacity to recover from the toxic effects of phlorotannins and phloroglucinol. This study showed that the toxic effects of phlorotannin and phloroglucinol depend on the concentration and time of exposure. The growth of *C. vulgaris* was significantly influenced by the concentration and exposure time to phlorotannins and phloroglucinol. A similar phenomenon was reported in previous studies, where the rate of inhibition of *C. vulgaris* increased during the initial period of exposure, but decreased at a later stage [53,55]. Microalgae are frequently exposed to toxic organic pollutants in aquatic ecosystems, which can disrupt the homeostasis of reactive oxygen species in cells. These species can act as a signal for cell activity and can also cause functional and structural damage to cells when they accumulate excessively, due to their strong oxidizing properties, which is the main mechanism of inhibition of microalgal growth. However, microalgae have protective mechanisms, such as increased photosynthetic pigments, carotenoids, biochemical characteristics, and antioxidant enzymes, which help species adapt to and protect against toxin-induced stress [54,67].

We observed that phlorotannins had a stronger inhibitory effect than phloroglucinol on *C. vulgaris* in both freshwater and seawater. After 96 h, the inhibition of approximately 39% of freshwater *C. vulgaris* by 2.0 mg/mL of phlorotannin was evident. The same level of inhibition was obtained with the use of phloroglucinol concentrations up to 5.0 mg/mL. In seawater *C. vulgaris*, the use of phlorotannin concentrations up to 2.0 mg/mL was able to inhibit the yield by approximately 43%, while the use of phloroglucinol concentrations of up to 5.0 mg/mL inhibited approximately 34% of treated populations. To the best of our knowledge, there have been no scientific reports on the inhibitory effect of phloroglucinol on *C. vulgaris*. It is presumed that *C. vulgaris* is more vulnerable to other compounds present in phlorotannins in addition to phloroglucinol. Further research is required on this topic. Lau and Qian [22] reported that 50% inhibition of the larval settlement of *H. elegans* occurred at a phlorotannin concentration of 13.98 ppm after 48 h, whereas 206.82 ppm phloroglucinol was required to obtain the same effect. 

Phlorotannin and phloroglucinol showed relatively stronger inhibition activities when tested on seawater *C. vulgaris* than on freshwater *C. vulgaris*. It is suspected that phlorotannin, which is a bioactive material from seaweed, is able to work more optimally in a seawater environment in accordance with its natural habitat. Beratto-Ramos et al. [68] reported that the structures that have the total polyphenolic content are affected by the environment. Additional studies are needed to investigate the effect of different environments on the phlorotannin and ploroglucinol toxicity level.

The level of larvicidal and inhibitory effect of crude phlorotannin extracted from *E. cava* appears to be relatively lower than crude phlorotannin from some other brown seaweed species. Many studies have reported that marine macroalgae in the *Phaeophyta* group contain a number of biodynamic compounds with their respective cytotoxic properties [16,17,19,57]. Phlorotannin, as a bio-compound of brown seaweed, may also have a different bioactivity and toxicity, depending on several factors, including structural composition and molecular weight [29,30,31,32]. Further research is needed to investigate this matter.

*E. cava* is found abundantly in Korea and has been used by East Asian people as edible marine brown algae as well as traditional herbal medicine. This phlorotannin toxicity study revealed that *E. cava* also has potential as a natural algaecide, pesticide, and herbicide agent for environmental management.

## 4. Conclusions

The brown seaweed *E. cava* obtained from coastal waters of South Korea has potential to be managed and utilized as a source of phlorotannins. Research on the environmental, biochemical, pharmaceutical, and other uses of phlorotannins extracted from brown seaweed is ongoing. Phlorotannins have larvicidal and inhibitory properties against various model organisms. Phloroglucinol, a phenol component of phlorotannin, has potential value due to its toxic properties. The use of *A. salina, D. magna, L. sativa*, and *C. vulgaris* in toxicity tests in the present study confirmed the larvicidal and inhibitory effects of various concentrations of phlorotannin and phloroglucinol. Phlorotannin and phloroglucinol were seen to be more larvicidal to *A. salina* than to *D. magna*, while the inhibitory effect was seen to be greater in seawater *C. vulgaris* than in freshwater *C. vulgaris*.

Results of this study are projected to complement information from previous similar studies and will inform future studies related to the toxicity of phlorotannins and other compounds derived from it. This research is also expected to provide basic data on the use of phlorotannins and phloroglucinol for application in divers fields, including aquaculture, as organic herbicides or larvicides.

## 5. Materials and Methods

### 5.1. Materials

#### 5.1.1. *Eclonia cava*

The *E. cava* was collected along the coast of South Korea in March–June 2021. *E. cava* with body lengths of 72–83 cm were selected, collected, and used in this study.

#### 5.1.2. Phloroglucinol

The phloroglucinol used for testing various organisms was a commercial product from ALDRICH Chemistry, with a purity of ≥99% (HPLC Grade).

#### 5.1.3. *Artemia salina* Nauplii

The eggs of dry brine shrimp (*A. salina*; SWORM, Seoul, Korea) were hatched in sterilized seawater with a 0.22 µm filter (1 g eggs per L) at 20 °C and lightly aerated under a constant light intensity of 40 mol/m^2^/s. Eggs were incubated in a glass flask (250 mL) at a water level of 20 cm. These hatching conditions corresponded to the natural environment of *A. salina*. *A. salina* nauplii were used for the experimental bioassays 24 h after hatching [26,69]. During the study period, nauplii still had yolk sacs. Therefore, no feeding was required. Nauplii are highly mobile and phototactic, making it easy to collect near light sources. The nauplii were collected using a Pasteur pipette and concentrated in small vials [26,70]. 

#### 5.1.4. *Daphnia magna* Neonates

The *D. magna* used in the experiment was selected from laboratory stock cultures. Adult *D. magna* were reared in a 2 L Erlenmeyer flask equipped with an aerator at 25 ± 1.0 °C and were fed *C. vulgaris* daily. Adult *D. magna* were reared to produce neonates. After 24 h, neonates were collected and used in the study [71,72,73].

#### 5.1.5. *Lactuca sativa* Seeds

Lettuce seeds (*L. sativa* L.; Korean cultivar, Hongbitjeokchimasangchu) were purchased from Danong Co., Ltd. (Namyangju, Korea). Seeds of uniform size were used.

#### 5.1.6. *Chlorella vulgaris*

The *C. vulgaris* was obtained from the Freshwater Bioresources Culture Collection, Republic of Korea (code FBCC-A49). Microalgae were divided into two parts. One part was cultured in freshwater, and the other was cultured in seawater 32 ppt. The use of the same *C. vulgaris* as an organism test for freshwater and seawater microalgae was intended so that the effects of phlorotannin and phloroglucinol on the inhibition of *C. vulgaris* could be compared equally. The *C. vulgaris* were cultured in the laboratory under aseptic conditions to avoid contamination. Microalgal were cultured in monocultures but were not axenic. The culture method was followed as described in previous studies [52,53,74]. The *C. vulgaris* was cultured in a 250 mL Erlenmeyer flask containing 150 mL Bold’s basal medium (BBM). The microalgal suspension was diluted with new sterilized BBM every 4 d to keep *C. vulgaris* in the exponential growth phase until it was used. During culture, *C. vulgaris* was placed in a model VS-8480 shaking incubator at 23.0 ± 1.0 °C and shaken at 150 rpm. The light intensity was set at 4000 lx, with a light:dark cycle of 14:10.

### 5.2. Methods

#### 5.2.1. Crude Phlorotannin Extracts 

The *E. cava* tissue was washed with tap water to remove salt, epiphytes, and sand and subsequently dried for 1 d at room temperature (24 ± 1 °C). The samples were then ground into a powder using a coffee grinder.

The methanol extraction procedure was carried out as previously described [24,26,28,75]. Algae powder (1 g) was mixed with methanol (4 mL) and shaken at room temperature for 2 h. Chloroform (CHCl_3_, 8 mL) was added to the solution, shaken vigorously by hand for 5 min, and filtered through defatted cotton or defatted filter paper. Distilled water (3 mL) was added to the solution and shaken by hand for 5 min. The supernatant was collected. The solution was extracted with diethyl ether (3 mL), and a layer was placed on top of the ether. This procedure was repeated twice. The final extract was evaporated using a nitrogen blower. Finally, the extract was dissolved in methanol and stored at −20 °C for later analysis. In the experiments, concentrations of phlorotannin extract were used to treat *A. salina* nauplii, *D. magna* neonates, *L. sativa* seeds, and *C. vulgaris*.

#### 5.2.2. Phloroglucinol 

The phloroglucinol profile of the crude phlorotannin was determined by high-performance liquid chromatography. The crude phlorotannin extract was separated in a C18 column (5 µm; 1 × 25 cm). The mobile phase was methanol and water with a gradient system from 30% to 100% of methanol for 40 min, followed by an isocratic system of 100% methanol for 5 min. The flow rate was adjusted to 1 mL/min, and the absorbance was monitored at 230 nm. 

Calibration plots of peak area (µVs) with standard phloroglucinol (0.1–2.0 mg/mL) provided the following equation:

y = 30,000,000x + 2,000,000

where y represents the peak area and x represents the phloroglucinol concentration.

#### 5.2.3. *Artemia salina* and *Daphnia magna* Lethality Test

A specific research system was designed to supply dissolved oxygen to each container in a 24-well plate containing a high density of *A. salina* nauplii or *D. magna* neonates (Supplementary Figure S1). Several infusion sets were cut at the base and compiled in a conical tube. The conical tube was connected to an air pump with a hose. To prevent the solution from overflowing, the 24 wells were coated with parafilm before being covered with a double lid to maintain the needles in stable positions. At the end of the infusion sets, the needles were inserted into each well through 0.6 mm diameter holes made in the lid using a mini electric grinder. A hose was provided using an aeration controller. Air bubbles were kept to a minimum to avoid the over-saturation of dissolved oxygen and to prevent the formation of large bubbles that could kill the organism.

The lethality assay procedure on *A. salina* and *D. magna* was slightly modified from previous descriptions [26,69,76]. This test was conducted to determine the toxic effects of phlorotannin extract and phloroglucinol on *A. salina* nauplii and *D. magna* neonates. Briefly, a 1000 L aliquot of seawater sterilized with a 0.22 m filter (for *A. salina*) or distilled water (for *D. magna*) was pipetted into a 24-well plate. *A. salina* or *D. magna* larvae solution (400 µL, containing 20 larvae) were added to each well. In determining the exposure concentration, we conducted a preliminary test. In the preliminary test, we applied several concentrations and observed the mortality of the test organism. Based on our preliminary test, the exposure concentration for phlorotannin extract was determined at 0.1, 1.0, 5.0, 10.0, and 50.0 mg/mL for *A. salina* and 0.5, 1.0, and 2.0 mg/mL for *D. magna*. Meanwhile, the exposure concentrations for phloroglucinol were 0.005, 0.01, 0.05, 0.1, and 0.5 mg/mL for both *A. salina* and *D. magna*. For *A. salina*, the preliminary test was conducted based on previous study. A study conducted by Kim and Choi [26] showed that A. *salina* mortality was >50% at a concentration of 25 mg/mL brown seaweed extract after 48 h. Hence, we increased the concentration to more than 25 mg/mL.

Sterilized seawater or fresh water was immediately added to bring the final well volume to 2.0 mL [26]. The toxicity was determined after 24, 48, and 72 h of exposure by counting the number of surviving larvae and calculating the mortality rate. A solution of 2.5% methanol and 0.22 µm filter-sterilized seawater (for *A. salina*) or distilled freshwater (for *D. magna*) was used as the vehicle control or negative control, respectively. The results are expressed as the average ± standard deviation (SD) of at least three replicates. Mortality was evaluated using previous criteria [26,77], with slight modifications. The larvae were considered dead if they showed no movement during the observation period. If the mortality rate was <50%, the extract concentration was considered non-larvicide. If the mortality rate was >50% but <75%, the extract concentration was considered mild larvicide. If the mortality rate exceeded 75%, the extract concentration was considered highly larvicidal. Finally, if 100% of the larvae died, the extract was considered extremely larvicidal.

#### 5.2.4. *Lactuca sativa* Germination Bioassays

One milliliter of each extract concentration (1, 10 and 50 mg/mL) was added to filter paper (55 mm diameter; Toyo Roshi Kaisha, Ltd., Tokyo, Japan) in sterile Petri dishes (6 cm diameter; SPL Life Science, Pocheon, Korea). Subsequently, the filter paper containing phlorotannin or phloroglucinol was completely dried on a clean bench, so the toxic compound could be absorbed perfectly on the filter paper. Ten uniformly sized seeds of *L. sativa* that had been sterilized were placed evenly on filter paper containing phlorotannin or phloroglucinol in each Petri dish. Then, they were moistened with 1.2 mL of polyoxyethylene sorbitan monolaurate (Tween 20, L33109, Duksan, Korea) at a concentration of 1% [28]. Tween 20 was used as a surfactant that is regarded to be nontoxic [28,78,79]. Tween 20 at a concentration of 1% was used as the vehicle control. All Petri dishes were stored in an incubator in the dark at 25 ± 1 °C. The germination rate was determined by counting the number of seeds that germinated at an interval of 24 h for 4 d. Root length was also recorded every 24 h during the observation period. Germination was considered to occur only after the radicle protruded by ≥1 mm [28,80]. Radicle length measurements were carried out manually using a digital caliper under sterile conditions. Germination percentages for each treatment were calculated and compared with controls given 1% of Tween 20 without phlorotannin or phloroglucinol. The germination percentage (%) was calculated as n/N × 100, where n is the number of germinated seeds and N is the number of seeds sown [28].

#### 5.2.5. Freshwater and Seawater *Chlorella vulgaris* Yield Inhibition Test

The test was slightly modified from previous descriptions [52,53]. The test was carried out by placing *C. vulgaris* in a 15 mL vial containing 15 mL of BBM medium with various concentrations of phlorotannin (0.5, 1.0, and 2.0 mg/mL) and phloroglucinol (50, 100, 500, 1000, and 5000 µg/mL). A control lacking phlorotannin or phloroglucinol was also used. Each control and treatment with *C. vulgaris* experiment had three replicates each. All vials were placed in an incubator under the same conditions as those used for maintenance. An aseptic technique was used to prevent contamination. The total exposure time was 72 h. The optical density (OD) of the algal suspension was monitored at 24, 48, and 72 h at 680 nm (OD_680_) using a SPECTROstar^®^ Nano spectrophotometer (BMG LABTECH, Offenburg, Germany). 

To determine the relationship between the OD_680_ and cell density (CD, cells/mL), algae with high cell densities were diluted to seven different concentrations. The OD_680_ value of each concentration was measured. The CD was then determined using a hemocytometer positioned in a microscope based on the Neubauer method. From the comparison between the OD value and the number of *C. vulgaris* cells, a linear relationship was obtained as follows:

CD = (OD_680_ + 0.0282)/0.0004) × 10,000 (R^2^ = 0.9936)

The relationship between OD_680_ and dry cell weight (DCW g/L) of *C. vulgaris* was determined based on previous studies [52,53]. The linear relationship was obtained as follows:

DCW = 0.1244 × OD_680_ + 0.017 (R^2^ = 0.9908)

The yield inhibition of *C. vulgaris* at each exposure to phlorotannin or phloroglucinol concentration was expressed as a percentage of the total DCW compared to controls. The yield inhibition rate (I_y_, %) was calculated using the following equation [53]:

% I_y_ = ((N_c_ − N_t_)/N_c_) × 100

where N_c_ represents DCW in the control group and N_t_ represents DCW in the treated groups.

#### 5.2.6. Statistical Analysis

All experiments were repeated at least three times. All data were tested for normality and homogeneity before the Student’s *t*-test analysis was carried out. The Student’s *t*-test was used to compare the treatment group with the control group. The results were considered statistically significant at *p* < 0.05. Probit analysis was used in *A. salina* and *D. magna* bioassays to obtain the LC50. The LC50 is the effective toxicant concentration capable of killing 50% of organisms in the toxicity test at the time of observation. In Probit analysis, a regression equation is obtained from the concentration of the solution exposed and the percentage of mortality of the test organism so that the LC50 value can be determined [20,39,57]. All statistical analyses were performed using Microsoft Excel.

## Figures and Tables

**Table 1:** Larvicidal effects of crude phlorotannin and phloroglucinol on survival of *Artemia salina* nauplii.

**Incubation Time (h)**	**Survival of *Artemia salina* Nauplii**						
	**Control**	**Vehicle (2.5% Methanol)**		**Crude Phlorotannin (mg/mL)**			
			0.1	1.0	5.0	10.0	50.0
24	100.0 ± 0.0	100.0 ± 0.0	100.0 ± 0.0	100.0 ± 0.0	100.00 ± 0.0	81.3 ± 7.5 *	0.0 ± 0.0 *
48	100.0 ± 0.0	100.0 ± 0.0	100.0 ± 0.0	100.0 ± 0.0	98.8 ± 2.5	80.0 ± 10.0 *	0.0 ± 0.0 *
72	90.0 ± 4.1	88.8 ± 2.5	83.8 ± 6.29	82.5 ± 2.9 *	85.0 ± 4.1	37.5 ± 6.5 *	0.0 ± 0.0 *
**Incubation Time (h)**	**Survival of *Artemia salina* Nauplii (%)**						
	**Control**		**Phloroglucinol (mg/mL)**				
			0.005	0.01	0.05	0.1	0.5
24	100.0 ± 0.0		100.0 ± 0.0	100.0 ± 0.0	68.3 ± 2.9 *	1.7 ± 2.9 *	0.0 ± 0.0 *
48	100.0 ± 0.0		100.0 ± 0.0	100.0 ± 0.0	48.3 ± 7.6 *	0.0 ± 0.0 *	0.0 ± 0.0 *
72	100.0 ± 0.0		96.7 ± 2.9	96.7 ± 5.8	31.7 ± 2.9 *	0.0 ± 0.0 *	0.0 ± 0.0 *

* significant difference; statistical significance was calculated using Student’s *t*-test and deemed statistically significant at *p* < 0.05, compared to the control. Values represent the average ± SD of measurements performed in at least triplicate, *n* = 20 ind/replicate.

**Table 2:** Larvicidal effects of crude phlorotannin and phloroglucinol on survival of *Daphnia magna* neonates.

**Incubation Time (h)**	**Survival of *D. magna* Neonates (%)**					
	**Control**	**Vehicle (2.5% Methanol)**	**Phlorotannin Extract (mg/mL)**			
			0.5	1.0	2.0	
24	95.0 ± 5.8	92.5 ± 5.0	91.3 ± 2.5	63.8 ± 6.3 *	16.3 ± 4.9 *	
48	87.5 ± 8.7	85.0 ± 7.1	38.8 ± 11.1 *	20.0 ± 12.2 *	0.0 ± 0.0 *	
72	87.5 ± 8.7	85.0 ± 7.1	31.3 ± 7.5 *	7.5 ± 6.5 *	0.0 ± 0.0 *	
**Incubation Time (h)**	**Survival of *Daphnia magna* Neonate (%)**					
	**Control**		**Phloroglucinol (mg/mL)**			
		0.005	0.01	0.05	0.1	0.5
24	96.7 ± 2.9	95.0 ± 0.0	98.3 ± 2.9	68.3 ± 2.9 *	28.3 ± 12.6 *	0.0 ± 0.0 *
48	93.3 ± 2.9	86.7 ± 2.9 *	83.3 ± 7.6 *	61.7 ± 5.8 *	25.0 ± 8.7 *	0.0 ± 0.0 *
72	93.3 ± 2.9	80.0 ± 5.0 *	73.3 ± 7.6 *	61.7 ± 2.9 *	16.7 ± 5.8 *	0.0 ± 0.0 *

* significant difference; statistical significance was calculated using Student’s *t*-test and deemed statistically significant at *p* < 0.05, compared to the control. Values represent the average ± SD of measurements performed in at least triplicate, *n* = 20 ind/replicate.

**Table 3:** Inhibitory effect of crude phlorotannin and phloroglucinol on germination of *Lactuca sativa* seed.

**Incubation Time (h)**	**Germination of *L. sativa* Seed (%) (Radicle Length of *L. sativa* Seed (mm))**					
	**Vehicle**	**Crude Phlorotannin (mg/mL)**				
		1.0		10.0		50.0
24	67.5 ± 9.8	40.0 ± 14.1 *		2.5 ± 5.0 *		2.5 ± 5.0 *
	(1.91 ± 0.75)	(0.93 ± 0.49)		(0.08 ± 0.15) *		(0.15 ± 0.29) *
48	85.0 ± 5.8	57.5 ± 9.6 *		37.5 ± 20.6 *		17.5 ± 17.1 *
	(9.04 ± 1.13)	(6.82 ± 1.32) *		(1.16 ± 0.63) *		(1.56 ± 1.86) *
72	90.0 ± 8.2	70.0 ± 8.2 *		57.5 ± 22.2% *		35.0 ± 19.2 *
	(17.78 ± 1.68)	(15.16 ± 2.75)		(2.06 ± 1.04) *		(4.24 ± 3.43) *
96	95.0 ± 5.8	70.0 ± 8.2 *		70.0 ± 11.6 *		52.5 ± 9.6 *
	(25.29 ± 2.39)	(22.33 ± 1.87)		(3.40 ± 1.98) *		(7.89 ± 4.86) *
**Incubation Time (h)**	**Germination of *L. sativa* Seed (%) (Radicle Length of *L. sativa* Seed (mm))**					
	**Vehicle**	**Phloroglucinol** **(µg/mL)**				
		50	100	500	1000	5000
24	46.7 ± 5.8	50.0 ± 24.5	50.0 ± 0.0	20.0 ± 17.3	0.0 ± 0.0 *	0.0 ± 0.0 *
	(2.05 ± 0.38)	(2.35 ± 1.07)	(2.21 ± 0.50)	(0.72 ± 0.57) *	(0.00 ± 0.00) *	(0.00 ± 0.00) *
48	83.3 ± 15.3	70.0 ± 20.0	73.3 ± 15.3	60.0 ± 0.0	13.3 ± 5.8 *	0.0 ± 0.0 *
	(10.82 ± 1.35)	(7.76 ± 2.97)	(7.24 ± 0.96) *	(4.21 ± 1.02) *	(0.39 ± 0.67) *	(0.00 ± 0.00) *
72	90.0 ± 10.0	76.7 ± 15.3	86.7 ± 5.8	63.3 ± 5.8 *	30.0 ± 10.0 *	0.0 ± 0.0 *
	(19.78 ± 1.10)	(13.83 ± 5.54)	(14.92 ± 2.04) *	(8.03 ± 0.37) *	(1.92 ± 0.97) *	(0.00 ± 0.00) *
96	96.7 ± 5.8	76.7 ± 15.3	86.7 ± 5.8	80.0 ± 0.0 *	53.3 ± 5.8 *	0.0 ± 0.0 *
	(29.78 ± 1.03)	(20.05 ± 7.83)	(21.89 ± 2.50) *	(11.73 ± 0.53) *	(3.53 ± 0.67) *	(0.00 ± 0.00) *

* significant difference; statistical significance was calculated using Student’s *t*-test and deemed statistically significant at *p* < 0.05, compared to the control. Values represent the average ± SD; all measurements were performed in at least triplicate, *n* = 10 ind/replicate.

**Table 4:** Toxicity of crude phlorotannin and phloroglucinol on cell density (CD) of freshwater *Chlorella vulgaris*.

**Exposure Time (h)**	**Cell Density (CD) of Freshwater *C. vulgaris* (10^6^ cells/mL)**					
	**Control**	**Crude Phlorotannin (mg/mL)**				
		0.5		1.0		2.0
0	1.22 ± 0.04	1.36 ± 0.08		1.33 ± 0.05		1.28 ± 0.07
24	1.46 ± 0.34	1.37 ± 0.08		1.49 ± 0.23		1.44 ± 0.28
48	2.31 ± 0.19	2.11 ± 0.10		1.82 ± 0.28		1.77 ± 0.18 *
72	3.41 ± 0.06	2.47 ± 0.15 *		1.89 ± 0.06 *		1.79 ± 0.11 *
96	4.80 ± 0.19	3.26 ± 0.18 *		2.24 ± 0.17 *		2.02 ± 0.12 *
**Exposure Time (h)**	**Cell Density (CD) of Freshwater *C. vulgaris* (10^6^ cells/mL)**					
	**Control**	**Phloroglucinol (µg/L)**				
		50	100	500	1000	5000
0	1.27 ± 0.02	1.24 ± 0.04	1.26 ± 0.10	1.22 ± 0.08	1.28 ± 0.07	1.30 ± 0.07
24	1.20 ± 0.08	1.12 ± 0.08	1.17 ± 0.08	1.11 ± 0.11	1.06 ± 0.09	1.12 ± 0.02
48	1.83 ± 0.08	1.61 ± 0.26	1.14 ± 0.05	1.23 ± 0.09	1.06 ± 0.03 *	1.03 ± 0.05 *
72	3.80 ± 0.09	3.35 ± 0.12 *	2.32 ± 0.09 *	1.78 ± 0.08 *	1.71 ± 0.06 *	1.47 ± 0.12 *
96	4.34 ± 0.17	3.44 ± 0.30 *	2.34 ± 0.18 *	1.82 ± 0.23 *	1.74 ± 0.19 *	1.57 ± 0.08 *

* significant difference; statistical significance was calculated using Student’s *t*-test and deemed statistically significant at *p* < 0.05, compared to the control. Values represent the average ± SD; all measurements were performed in at least triplicate.

**Table 5:** Toxicity of crude phlorotannin and phloroglucinol on cell density (CD) of seawater *Chlorella vulgaris*.

**Exposure Time (h)**	**Cell Density (CD) of Seawater *C. vulgaris* (10^6^ cells/mL)**					
	**Control**	**Crude Phlorotannin (mg/mL)**				
		0.5	1.0	2.0		
0	1.41 ± 0.07	1.35 ± 0.13	1.40 ± 0.17	1.25 ± 0.22		
24	2.76 ± 0.05	2.64 ± 0.09	2.30 ± 0.02 *	1.51 ± 0.06 *		
48	3.72 ± 0.09	3.31 ± 0.09 *	1.55 ± 0.06 *	1.31 ± 0.05 *		
72	3.83 ± 0.10	3.47 ± 0.05 *	1.77 ± 0.04 *	1.18 ± 0.08 *		
96	4.47 ± 0.06	4.01 ± 0.08 *	2.32 ± 0.09 *	1.33 ± 0.07 *		
**Exposure Time (h)**	**Cell Density (CD) of Seawater *C. vulgaris* (10^6^ cells/mL)**					
	**Control**	**Phloroglucinol (µg/L)**				
		50	100	500	1000	5000
0	1.26 ± 0.07	1.29 ± 0.08	1.26 ± 0.02	1.24 ± 0.04	1.21 ± 0.07	1.19 ± 0.06
24	1.62 ± 0.07	1.67 ± 0.01	1.50 ± 0.03 *	1.47 ± 0.07 *	1.37 ± 0.03 *	1.33 ± 0.03 *
48	1.86 ± 0.07	1.69 ± 0.04 *	1.68 ± 0.05 *	1.35 ± 0.06 *	1.21 ± 0.07 *	1.14 ± 0.02 *
72	3.44 ± 0.11	3.03 ± 0.07 *	2.79 ± 0.06 *	1.94 ± 0.07 *	1.78 ± 0.08 *	1.32 ± 0.04 *
96	4.14 ± 0.16	3.32 ± 0.12 *	2.97 ± 0.17 *	2.52 ± 0.11 *	2.42 ± 0.05 *	1.80 ± 0.16 *

* significant difference; statistical significance was calculated using Student’s *t*-test and deemed statistically significant at *p* < 0.05, compared to the control. Values represent the average ± SD; all measurements were performed in at least triplicate.

## Data Availability

Data supporting reported results are available upon request.

## Supplementary Materials

The following supporting information can be downloaded at: https://www.mdpi.com/article/10.3390/toxins14050312/s1, Figure S1: Design of the system used to assess the larvicidal effect of concentrations of phlorotannin and phloroglucinol on *Artemia salina* nauplii and *Daphnia magna* neonates.
