# Supplementary material for: Evaluation of Toxicity of Crude Phlorotannins and Phloroglucinol Using Different Model Organisms
Source: Toxins (Basel). 2022 Apr 28;14(5):312. doi: 10.3390/toxins14050312 (PMC9148043; doi:10.3390/toxins14050312)
Supplement: Supplementary file 1 [file toxins-14-00312-s001.zip › toxins-1666693-supplementary.pdf]

Supplementary Materials: Evaluation of Toxicity of Crude Phlorotannins and Phloroglucinol Using Different Model Organisms

Dicky Harwanto, Bertoka Fajar Surya Perwira Negara, Gabriel Tirtawijaya, Maria Dyah Nur Meinita and Jae-Suk Choi

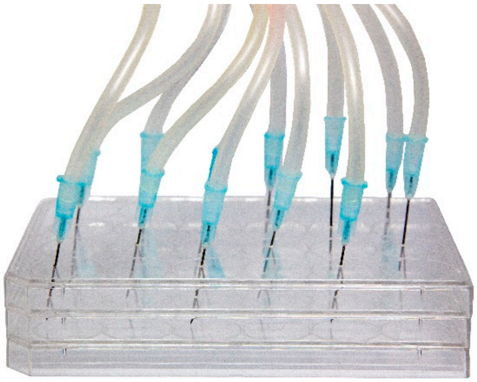

Figure S1. Design of the system used to assess the larvicidal effect of concentrations of phlorotannin and phloroglucinol on *Artemia salina* nauplii and *Daphnia magna* neonates.
